# Environmental monitoring of soil carbon and ecosystem productivity across land use systems in the Atlantic Forest of Brazil

**DOI:** 10.1007/s10661-026-15952-4

**Published:** 2026-09-23

**Authors:** Romario Oliveira de Santana, Lídia Raíza Sousa Lima Chaves Trindade, Rafael Coll Delgado

**Affiliations:** 1 Coordination for the Creation of Conservation Units (COCUC), Chico Mendes Institute for Biodiversity Conservation (ICMBio), Federal District, Brasília, 70680-400 Brazil; 2 Institute of Mathematics and Statistics, University of São Paulo (USP), São Paulo, São Paulo 05508-090 Brazil; 3 Center for Biological and Natural Sciences, Federal University of Acre (UFAC), Rio Branco, Acre 23897-000 Brazil

**Keywords:** Carbon dynamics, Environmental conservation, Geoprocessing, Land-use change, Vegetation productivity

## Abstract

Tropical ecosystems play a fundamental role in the global carbon cycle due to their high productivity and capacity to store carbon in vegetation and soils. However, land use change has substantially altered these processes, particularly in highly fragmented regions such as the Central Atlantic Forest Corridor (CAFC), one of the major biodiversity hotspots in Brazil. This study analyzes the relationship between gross primary productivity (GPP), soil organic carbon (SOC), land use, and soil texture to identify spatial patterns and functional regimes across the landscape. GPP data derived from MODIS, SOC stocks from MapBiomas Soil, and land use and land cover data were integrated using geoprocessing and statistical analyses. Significant differences were observed among land use classes, with higher GPP in forest ecosystems and higher SOC stocks in natural nonforest formations. The relationship between GPP and SOC was predominantly weak, indicating a decoupling between contemporary productivity and soil carbon storage. Both variables exhibited significant spatial autocorrelation, and after accounting for spatial dependence, the effect of GPP on SOC was weak and temporally inconsistent, reinforcing this decoupling. The integrated spatial analysis identified distinct functional regimes, with ecological hotspots concentrated predominantly in forest ecosystems and low-functionality regimes strongly associated with pasturelands. These findings demonstrate that ecosystem productivity and soil carbon storage exhibit distinct spatial and functional responses across the landscape. Integrating these complementary dimensions of ecosystem carbon functioning can support conservation, ecological restoration, and carbon-oriented environmental planning in fragmented tropical landscapes.

## Introduction

The global carbon cycle is regulated by continuous exchanges among the atmosphere, vegetation, soils, and oceans. Among terrestrial ecosystems, tropical regions stand out due to their high biological productivity and their capacity to store large amounts of carbon in both biomass and soils, thereby playing a crucial role in regulating the Earth’s climate (Beer et al., 2010; Friedlingstein et al., 2026). However, land use change and global warming have altered the dynamics of these carbon stocks, increasing the importance of studies aimed at understanding the ecological processes that control carbon storage in tropical landscapes (Bossio et al., 2020; IPCC, 2021).

Although vegetation is often associated with atmospheric carbon sequestration, soils represent one of the largest carbon reservoirs on Earth, storing more carbon than the atmosphere and plant biomass combined (Jobbágy & Jackson, 2000; Lal, 2004). Soil Organic Carbon (SOC) performs essential functions in soil fertility, water retention, physical structure, and ecosystem stability, while also serving as a key component of climate change mitigation (Lehmann & Kleber, 2015; Schmidt et al., 2011). However, the maintenance of these carbon stocks depends on multiple environmental controls, including climate, soil texture, hydrological regime, microbial activity, and land-use management history (Bossio et al., 2020; Lal, 2004).

The primary pathway for carbon input into soils is through plant production. In this context, gross primary productivity (GPP), defined as the total amount of carbon assimilated through photosynthesis, is one of the most important indicators of ecosystem functioning and terrestrial carbon sequestration capacity (Beer et al., 2010; Running & Zhao, 2021). A portion of this carbon is transferred to the soil through litterfall, root growth, root exudates, and biomass turnover (Chapin et al., 2006). Nevertheless, the relationship between GPP and SOC is not necessarily direct, since soil carbon stocks reflect long-term cumulative processes mediated by decomposition, organo-mineral protection, nutrient availability, and environmental disturbances (Cotrufo et al., 2019; Schmidt et al., 2011; Terrer et al., 2021). Consequently, highly productive areas do not always correspond to areas with the highest soil carbon stocks (Lehmann & Kleber, 2015).

This complexity becomes even more relevant in tropical biomes undergoing intense anthropogenic transformation, such as the Atlantic Forest. Recognized as one of the world’s major biodiversity hotspots, the Atlantic Forest combines exceptional biological richness and high levels of endemism with a long history of fragmentation and conversion of native vegetation (Myers et al., 2000; Ribeiro et al., 2009). Originally covering approximately 1.3 million km^2^, the Atlantic Forest has undergone extensive historical loss and fragmentation of its native vegetation as a result of centuries of human occupation and land use change, leaving its remaining forest embedded within highly modified landscapes (Rezende et al., 2018).

Within this context, the Central Atlantic Forest Corridor (CAFC) represents an important landscape-scale conservation initiative aimed at promoting ecological connectivity among Atlantic Forest remnants across southern Bahia and Espírito Santo (Santana et al., 2022). The region is characterized by high environmental heterogeneity and a complex land use mosaic, including native forests, cocoa agroforestry systems (cabruca), commercial forest plantations, intensive agriculture, and extensive pasturelands (Santana et al., 2022; Schroth et al., 2011). This spatial configuration directly influences carbon fluxes, vegetation productivity, and the soil carbon storage capacity.

Recent advances in remote sensing and cloud-based spatial modeling have substantially enhanced the ability to monitor vegetation productivity and carbon stocks at regional scales. MODIS-derived GPP products provide spatially explicit estimates of terrestrial productivity (Running & Zhao, 2021), while national initiatives such as MapBiomas Soil provide spatially explicit estimates of SOC stocks across Brazil (MapBiomas, 2025). Nevertheless, the joint assessment of GPP and SOC across contrasting land use systems is important for understanding whether ecosystem productivity and belowground carbon storage exhibit convergent or contrasting patterns in fragmented tropical landscapes (Schmidt et al., 2011; Terrer et al., 2021). Integrating these complementary dimensions of the terrestrial carbon cycle with soil properties and land use information can provide a more comprehensive basis for characterizing ecosystem functioning and informing conservation and ecological restoration strategies (Bossio et al., 2020).

Therefore, the present study aims to spatially model the relationship between gross primary productivity and soil organic carbon in the Central Atlantic Forest Corridor, considering the influence of land-use and land cover as well as soil texture. Specifically, the study seeks to address the following questions: (i) How do GPP and SOC vary among different land use classes? (ii) Is the relationship between ecosystem productivity and soil carbon stocks consistent after accounting for spatial dependence? (iii) What spatial patterns and functional regimes emerge from the interaction between these variables? (iv) How can these spatial patterns contribute to regional conservation, ecological restoration, and carbon-oriented environmental planning?

## Materials and methods

### Study area

**Fig. 1:**
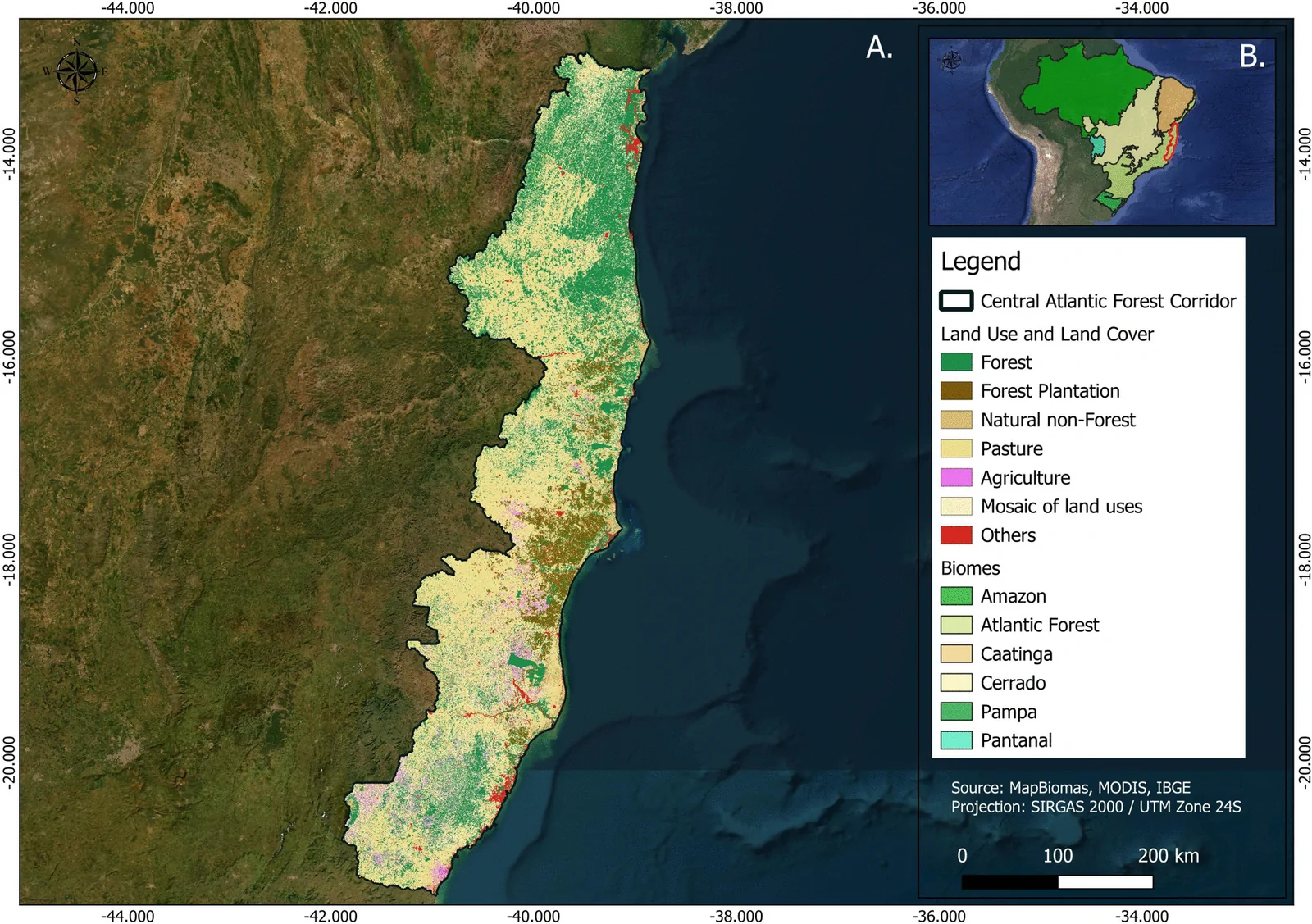
Location of the Central Atlantic Forest Corridor (CAFC), Brazil, highlighting its regional setting within the context of the Brazilian biomes. **A** The spatial extent of the study area and the overall distribution of the aggregated land use and land cover classes. **B** The location of the CAFC within the Brazilian territory and its relationship with the country’s major biomes

The study was conducted in the Central Atlantic Forest Corridor (CAFC) (Fig. 1), located in eastern Brazil and encompassing areas within the states of Bahia and Espírito Santo, between latitudes 13°00′06″ and 21°18′36″ S and longitudes 41°52′43″ and 37°16′40″ W. The corridor covers approximately 13.3 million hectares and comprises a heterogeneous landscape of Atlantic Forest remnants and different land use and land cover types (Santana et al., 2022).

Within the CAFC, there are 258 protected areas distributed across private and public domains at the federal, state, and municipal levels and encompassing different management categories (Santana et al., 2022). These protected areas are organized under the Brazilian National System of Conservation Units (SNUC; Law No. 9,985/2000) into two broad groups: strict protection and sustainable use.

The region exhibits a pronounced environmental gradient between the Atlantic coastal zone and inland sectors, reflected in climatic, topographic, and ecological variation. Tropical climates predominate across the CAFC, including areas without a dry season (Af) and areas characterized by a dry winter (Aw) (Alvares et al., 2013; Santana et al., 2022).

### Data and processing

Annual data on gross primary productivity (GPP) and soil organic carbon (SOC) were analyzed for the period from 2001 to 2023.

GPP was obtained from the MODIS/Terra MOD17A3HGF Version 6.1 (V061) product, which provides annual estimates of Gross Primary Productivity at a spatial resolution of 500 m (Running & Zhao, 2021). The product provides annual gap-filled estimates of GPP derived from MODIS observations and associated biophysical inputs. GPP was expressed in kg C m⁻^2^ yr⁻^1^ (Running & Zhao, 2021).

SOC data were obtained from the MapBiomas Soil Collection 3 (Beta), which provides annual maps of Soil Organic Carbon stocks in the 0–30-cm soil layer across Brazil at a spatial resolution of 30 m (MapBiomas, 2025). SOC stocks are expressed in t ha⁻^1^ and were estimated using a spatiotemporal machine learning framework based on field soil observations and environmental covariates (Samuel-Rosa et al., 2025).

Land use and land cover (LULC) data were obtained from the MapBiomas Annual Land Use and Land Cover Mapping Series, Collection 10.1, at a spatial resolution of 30 m (MapBiomas, 2026). The MapBiomas mapping framework uses Landsat imagery and cloud-based processing to generate annual LULC classifications across Brazilian biomes (Souza et al., 2020). For analytical purposes, the original classes were aggregated into six categories: forest (natural forest formation), natural nonforest formation, agriculture, pasture, forestry plantations, and land use mosaic.

To ensure spatial consistency among variables, all datasets were resampled to a common 500-m grid, matching the spatial resolution of the MODIS product. Temporal harmonization was based on annual composites, with each pixel assigned to the corresponding land use class for the respective year.

Initial spatial processing, including clipping the study area, spatial resolution standardization, data extraction, and organization of the time series, was performed using Google Earth Engine (Gorelick et al., 2017). The resulting datasets were subsequently organized and analyzed in the R programming language (R Core Team, 2024).

### Statistical analysis

The distributions of GPP and SOC were initially evaluated using the Shapiro–Wilk test for normality and Levene’s test for homogeneity of variances, respectively (Levene, 1960; Shapiro & Wilk, 1965). Both variables exhibited significant departures from normality and homogeneity of variances (*p* < 0.001). Therefore, comparisons of GPP and SOC among land use classes were conducted using nonparametric procedures.

Differences among land use classes were assessed using the Kruskal–Wallis test, followed by Dunn’s multiple comparison test with Bonferroni correction (Dunn, 1964; Kruskal & Wallis, 1952). These procedures were specifically applied to group comparisons and allowed the identification of statistically significant differences among the analyzed land use classes.

The use of nonparametric procedures for comparisons among land use classes was distinguished from the regression-based analyses. The latter were used to quantify conditional associations among GPP, SOC, soil clay content, and land use rather than to compare the marginal distributions of the variables. Because the observations were spatially structured, the assumption of spatial independence was explicitly evaluated using spatial autocorrelation diagnostics and subsequently addressed through spatial regression models, as described in the corresponding section.

Data distribution and variability were additionally examined using box plots combined with random subsampling (jitter), allowing visualization of data dispersion and extreme values, as well as histograms to characterize distribution shapes and skewness. All statistical analyses were performed in the R programming language, primarily using the tidyverse, ggplot2, rstatix, trend, and car packages.

### Relationship between GPP and SOC

The relationship between gross primary productivity and soil organic carbon was evaluated using simple linear regression models fitted separately for each land use class, according to the following equation:

1 $$\mathrm{S}\mathrm{O}\mathrm{C}={\beta}_{0}+{\beta}_{1}\mathrm{G}\mathrm{P}\mathrm{P}+\varepsilon$$

where SOC represents soil organic carbon, GPP represents gross primary productivity, *β*₀ is the intercept, *β*₁ is the regression coefficient representing the estimated change in SOC associated with a one-unit change in GPP, and ε is the residual error term.

Model performance was evaluated using the coefficient of determination (*R*^2^) and statistical significance levels, allowing the degree of coupling between ecosystem productivity and soil carbon storage to be assessed across different land use systems (Montgomery et al., 2021).

### Multivariate modeling

To evaluate the factors controlling the variability of SOC and GPP, multiple linear regression models were fitted.

For SOC, the model included GPP, soil clay content (%), and land use classes as explanatory variables:

2 $$\mathrm{S}\mathrm{O}\mathrm{C}={\beta}_{0}+{\beta}_{1}\mathrm{G}\mathrm{P}\mathrm{P}+{\beta}_{2}\mathrm{C}\mathrm{l}\mathrm{a}\mathrm{y}+\sum_{\left(k=1\right)}^{\left(K-1\right)}{\gamma}_{k}L{U}_{k}+\varepsilon$$

where SOC represents soil organic carbon, GPP represents gross primary productivity, Clay represents soil clay content (%), *β*₀ is the intercept, *β*₁ and *β*₂ are the regression coefficients associated with GPP and clay content, respectively, *LUₖ* represents the dummy variable for the *k*th land use class, *γₖ* is the corresponding land use coefficient, *K* is the total number of land use classes, and ε is the residual error term.

For GPP, the variables considered were land use and clay content:

3 $$\mathrm{G}\mathrm{P}\mathrm{P}={\beta}_{0}+{\beta}_{1}\mathrm{C}\mathrm{l}\mathrm{a}\mathrm{y}+\sum_{\left(k=1\right)}^{\left(K-1\right)}{\gamma}_{k}L{U}_{k}+\varepsilon$$

where GPP represents gross primary productivity, Clay represents soil clay content (%), *β*₀ is the intercept, *β*₁ is the regression coefficient associated with clay content, *LUₖ* represents the dummy variable for the *k*th land use class, *γₖ* is the corresponding land use coefficient, *K* is the total number of land use classes, and ε is the residual error term.

Land use classes were treated as categorical variables. Model performance was evaluated using the coefficient of determination (*R*^2^), the statistical significance of the regression coefficients, and multicollinearity diagnostics based on the variance inflation factor (VIF) (James et al., 2021). All modeling procedures were performed in the R programming language.

### Temporal analysis

Temporal trends in GPP and SOC were assessed using the Mann–Kendall test, which is appropriate for nonparametric time series analysis (Kendall, 1975; Mann, 1945). The magnitude and direction of the trends were estimated using Sen’s slope estimator (Sen, 1968):

4 $$\beta =\mathrm{m}\mathrm{e}\mathrm{d}\mathrm{i}\mathrm{a}\mathrm{n}\left(\left({x}_{j}-{x}_{i}\right)/\left(j-i\right)\right)$$

where *β* represents Sen’s slope estimate, ${x}_{i}$ and ${x}_{j}$ are the observed values at time points *i* and *j*, respectively, and *j* − *i* represents the time interval between the two observations.

The analyses were conducted both at the regional scale and by land use class, allowing the identification of distinct temporal dynamics across different land use systems.

### Classification of GPP and SOC hotspots

Spatial patterns associated with the interaction between gross primary productivity and soil organic carbon were identified using a combined classification based on GPP and SOC values. Global median values for each variable, calculated from the complete dataset, were used as threshold values.

Each observation was classified into one of four groups according to the combination of values above or below these thresholds, as defined by the following function:

5 $$\mathrm{Hotspot}\;(\mathrm{SOC},\mathrm{GPP})=\left\{\begin{array}{cc}\mathrm{Low}\;\mathrm{SOC}+\mathrm{Low}\;\mathrm{GPP}&\mathrm{SOC}<{\mathrm{Med}}_{\mathrm{SOC}}\,\;\mathrm{and}\,\mathrm{GPP}<{\mathrm{Med}}_{\mathrm{GPP}}\\\mathrm{Low}\;\mathrm{SOC}+\mathrm{High}\;\mathrm{GPP}&\mathrm{SOC}<{\mathrm{Med}}_{\mathrm{SOC}}\;\,\mathrm{and}\,\mathrm{GPP}\geq{\mathrm{Med}}_{\mathrm{GPP}}\\\mathrm{High}\;\mathrm{SOC}+\mathrm{Low}\;\mathrm{GPP}&\mathrm{SOC}\geq{\mathrm{Med}}_{\mathrm{SOC}}\;\mathrm{and}\;\mathrm{GPP}<{\mathrm{Med}}_{\mathrm{GPP}}\\\mathrm{High}\;\mathrm{SOC}+\mathrm{High}\;\mathrm{GPP}&\mathrm{SOC}\geq{\mathrm{Med}}_{\mathrm{SOC}}\,\mathrm{and}\,\mathrm{GPP}\geq{\mathrm{Med}}_{\mathrm{GPP}}\end{array}\right.$$

where SOC represents soil organic carbon, GPP represents gross primary productivity, and ${\mathrm{M}\mathrm{e}\mathrm{d}}_{\mathrm{S}\mathrm{O}\mathrm{C}}$ and ${\mathrm{M}\mathrm{e}\mathrm{d}}_{\mathrm{G}\mathrm{P}\mathrm{P}}$ represent the global median values of SOC and GPP, respectively, used as thresholds for the classification of the four functional regimes.

This approach enables the identification of distinct functional regimes across the landscape by jointly considering ecosystem productivity and soil carbon storage. Based on the combinations of GPP and SOC values, four functional regimes were defined: areas with simultaneously low values for both variables, areas dominated by either productivity or soil carbon storage, and ecological hotspots characterized by the co-occurrence of high GPP and SOC values.

The median of each variable was used as the classification threshold because of the asymmetric distribution of the data, providing greater robustness against the influence of extreme values. Accordingly, pixels were classified according to whether their GPP and SOC values were above or below their respective medians. For cartographic representation and improved visual interpretation of the results, the resulting analytical classes were assigned descriptive categories while maintaining direct correspondence with the original combinations of GPP and SOC. Table 1 presents the equivalence between the analytical nomenclature and the descriptive terms used in the map legends.

**Table 1:** Correspondence between the analytical hotspot classification and the nomenclature used in the cartographic representation

Analytical classification	Functional interpretation	Map legend
Low SOC + low GPP	Low ecological performance	Low functionality
Low SOC + high GPP	High productivity with low carbon stock	Productivity-dominated
High SOC + low GPP	High carbon stock with low productivity	Carbon-dominated
High SOC + high GPP	High productivity and high carbon stock	Ecological hotspot

Additionally, a contingency table was constructed between the hotspot classes and the land use groups to evaluate the distribution of these functional regimes across the landscape.

For interpretation and visualization of the association patterns, the contingency table values were normalized by row and expressed as the proportion (%) of each land use class within each functional regime. These results were displayed using a heatmap, enabling the identification of dominant patterns of association between ecological regimes and land use.

The statistical significance of the association between functional regimes and land use classes was evaluated using the chi-square test of independence (*χ*^2^), applied to the contingency table of observed frequencies. The test was performed under the null hypothesis (H₀) that the variables were independent and the alternative hypothesis (H₁) that an association existed between functional regimes and land use.

The chi-square (*χ*^2^) statistic was calculated based on the difference between observed and expected frequencies, according to the following equation:

6 $${\chi }^{2}=\sum \frac{\left({O}_{i}-{E}_{i}{)}^{2}\right.}{{E}_{i}}$$

where ${O}_{i}$ represents the observed frequencies and ${E}_{i}$ represents the expected frequencies under the null hypothesis of independence (Agresti, 2019).

### Spatial autocorrelation and spatial regression

Spatial dependence in GPP and SOC was evaluated using Global Moran’s *I*, a widely used measure of global spatial autocorrelation that quantifies the degree of similarity among observations according to their spatial arrangement (Cliff & Ord, 1981; Moran, 1950). Because the dataset consisted of repeated annual observations at fixed sampling locations, spatial autocorrelation was assessed separately for each year from 2001 to 2023, preserving the annual spatial structure and avoiding the treatment of repeated observations from the same locations as independent spatial units.

Prior to spatial analysis, geographic coordinates were projected to the SIRGAS 2000/UTM Zone 24S coordinate reference system (EPSG:31984). Spatial relationships among sampling locations were defined using a *k*-nearest-neighbor approach, adopting the eight nearest neighbors (*k* = 8) as the primary spatial connectivity criterion. This approach defines spatial relationships according to proximity while ensuring that each observation has a predefined number of neighbors. The resulting spatial weights matrix was row-standardized. Statistical significance of Global Moran’s *I* was assessed using 999 random permutations.

Global Moran’s *I* was calculated according to

7 $$I=\frac{n}{{S}_{0}}\frac{\sum_{i}\sum_{j}{w}_{ij}\left({x}_{i}-\overline{x }\right)\left({x}_{j}-\overline{x }\right)}{\sum_{i}{\left({x}_{i}-\overline{x }\right)}^{2}}$$

where *n* is the number of spatial observations, *wᵢⱼ* represents the spatial weight between observations *i* and *j*, *x*ᵢ and *x*ⱼ are the observed values, is the mean of the variable, and *S*₀ is the sum of all spatial weights (Moran, 1950).

Spatial autocorrelation was initially evaluated for GPP and SOC and subsequently assessed in the residuals of the multiple linear regression models. Residual spatial autocorrelation may violate the independence assumption of conventional regression models and affect statistical inference when the spatial structure of the data is not explicitly considered (Anselin, 1988; Dormann et al., 2007). Therefore, spatial error models (SEMs) were fitted separately for each year to account for spatial dependence remaining in the error structure. The SEM specification followed the spatial regression framework proposed by Anselin (1988):

8 $$y=X\beta +u,\hspace{1em}u=\lambda Wu+\varepsilon$$

where *y* is the response variable, *X* is the matrix of explanatory variables, *β* is the vector of regression coefficients, *W* is the row-standardized spatial weights matrix, *λ* is the spatial error coefficient, *u* represents the spatially structured error component, and *ε* represents the remaining random error (Anselin, 1988; Bivand et al., 2013).

For SOC, the spatial model included GPP, soil clay content, and land use class as explanatory variables, whereas the GPP model included soil clay content and land use class. The spatial models therefore maintained the same explanatory structure as the corresponding nonspatial multiple regression models, allowing changes in the magnitude and statistical significance of the estimated relationships to be evaluated after accounting for spatial dependence.

Model performance was evaluated by comparing regression coefficients, statistical significance, Akaike’s information criterion (AIC), and residual Moran’s *I* between the nonspatial and spatial models. Lower AIC values were interpreted as indicating greater relative support among models fitted to the same response data (Burnham & Anderson, 2002). The reduction in residual Moran’s *I* was additionally used to assess whether the SEM adequately accounted for the spatial dependence remaining in the nonspatial models.

To evaluate the sensitivity of the results to the specification of spatial connectivity, alternative spatial weight matrices based on four (*k* = 4) and twelve (*k* = 12) nearest neighbors were also examined, while the eight-neighbor structure (*k* = 8) was retained as the primary specification. Consistency in the direction and magnitude of the main effects across these alternative neighborhood definitions was used as an assessment of model robustness.

Spatial analyses were conducted in the R environment using the sf package for spatial data handling (Pebesma, 2018), spdep for the construction of spatial weights and spatial autocorrelation analysis (Bivand & Wong, 2018), and spatialreg for spatial regression modeling (Bivand et al., 2013).

## Results

### Differences in GPP and SOC among land use classes

**Fig. 2:**
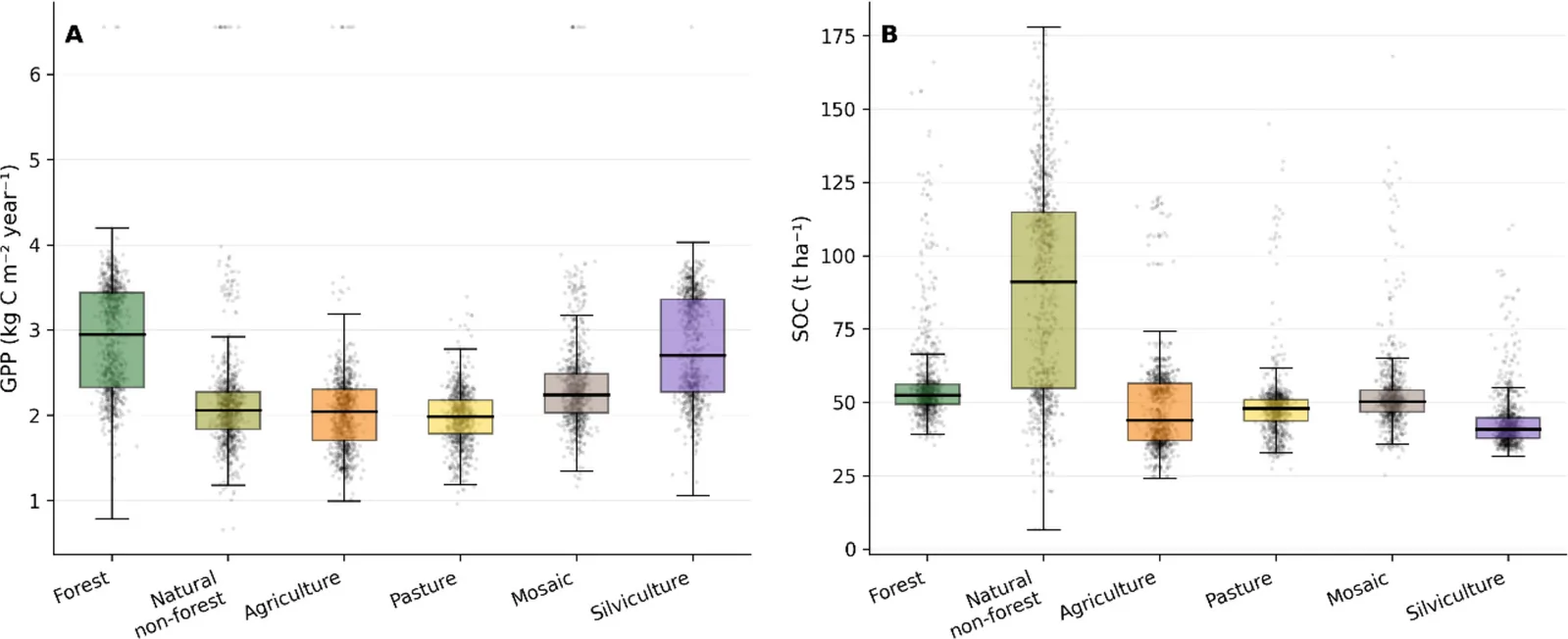
Differences in gross primary productivity (GPP) and soil organic carbon (SOC) among land use classes. **A** Distribution of gross primary productivity (GPP). **B** Distribution of soil organic carbon (SOC). Boxes represent the median and interquartile range (IQR), with whiskers indicating data variability. Points represent a random subsample used to visualize data dispersion

Gross primary productivity (GPP) and soil organic carbon (SOC) exhibited marked differences among land use and land cover classes, highlighting the fundamental role of land use in shaping the ecological processes analyzed (Table 2, Fig. 2). The distribution of GPP across land use classes revealed a well-defined functional hierarchy, in which forest systems exhibited the highest values and substantial variability, indicating not only high average productivity but also considerable internal heterogeneity within these ecosystems.

**Table 2:** Descriptive statistics (mean ± standard deviation) of gross primary productivity (GPP) and soil organic carbon (SOC) by land use class

Class	GPP (kg C m⁻^2^ yr⁻^1^)	SOC (t ha⁻^1^)
Forest	2.89 ± 0.63	56.0 ± 14.4
Forestry plantations	2.78 ± 0.60	42.7 ± 8.43
Land use mosaic	2.35 ± 0.63	52.4 ± 13.3
Natural nonforest formation	2.17 ± 0.75	88.3 ± 34.1
Agriculture	2.04 ± 0.52	47.8 ± 15.0
Pasture	2.00 ± 0.41	48.0 ± 10.4

The highest mean GPP values were observed in the forest (natural forest formation) and forestry plantation classes, with averages of 2.89 ± 0.63 and 2.78 ± 0.60 kg C m⁻^2^ yr⁻^1^, respectively. Figure 2A shows that natural forests exhibit a broad range of variation, whereas forestry plantations, despite their high productivity, display lower variability, suggesting greater structural homogeneity compared with natural forests.

In contrast, agriculture (2.04 ± 0.52 kg C m⁻^2^ yr⁻^1^) and pasture (2.00 ± 0.41 kg C m⁻^2^ yr⁻^1^) exhibited the lowest productivity levels, with more compact distributions, reflecting structural limitations associated with simplified vegetation cover. The land use mosaic showed intermediate behavior, whereas natural nonforest formations exhibited relatively low productivity values, similar to those observed in agricultural systems, indicating that high productivity is not a generalized characteristic of all natural ecosystems.

For SOC, the observed pattern differs substantially from that identified for GPP (Table 2, Fig. 2B). Natural nonforest formations exhibited the highest carbon stocks (88.3 ± 34.1 t ha⁻^1^) and the greatest variability, indicating environments with a high capacity for carbon accumulation and pronounced spatial heterogeneity.

Natural forests showed intermediate values (56.0 ± 14.4 t ha⁻^1^), followed by the land use mosaic (52.4 ± 13.3 t ha⁻^1^), whereas agriculture, pasture, and forestry plantations exhibited the lowest carbon stocks and more homogeneous distributions. This contrast demonstrates that the highest soil carbon stocks are not necessarily associated with the most productive ecosystems, suggesting that additional factors regulate soil carbon accumulation.

The differences among land use classes were statistically robust (Table 3). The Kruskal–Wallis test revealed highly significant differences for both GPP (*χ*^2^ = 144.838; *p* < 0.001) and SOC (*χ*^2^ = 83.558; *p* < 0.001). Dunn’s post hoc multiple comparisons indicated significant differences among nearly all pairwise comparisons, with the exception of SOC between agriculture and pasture, which did not differ significantly. Taken together, these findings indicate that although both variables are strongly influenced by land use, their spatial patterns are not equivalent, revealing a partial decoupling between ecosystem productivity and soil carbon storage.

**Table 3:** Results of nonparametric tests for GPP and SOC across land use classes

Variable	Test	Test statistic (*χ*^2^)	*p* value	Multiple comparisons (Dunn)
GPP	Kruskal–Wallis	144.838	< 0.001	Significant differences among all land use classes
SOC	Kruskal–Wallis	83.558	< 0.001	Significant differences among all land use classes, except agriculture vs. pasture

### Distribution and variability of GPP and SOC

The distributions of gross primary productivity (GPP) and soil organic carbon (SOC) reveal distinct patterns of variability across the study area, reflecting different ecological control mechanisms. GPP is predominantly concentrated between approximately 1.5 and 3.0 kg C m⁻^2^ yr⁻^1^, with a slight right-skewed distribution and relatively few high-value observations, indicating that the landscape is dominated by moderate productivity levels, with localized contributions from highly productive areas.

In contrast, SOC exhibits a more strongly skewed distribution, with most values ranging between approximately 30 and 70 t ha⁻^1^ and a long upper tail associated with high carbon stocks. Although these extreme values are relatively uncommon, they exert a substantial influence on the descriptive statistics, reflecting the pronounced spatial heterogeneity of soil carbon stocks. This pattern suggests that carbon accumulation is more strongly influenced by site-specific environmental conditions, such as soil properties and land use history, than by current ecosystem productivity.

**Fig. 3:**
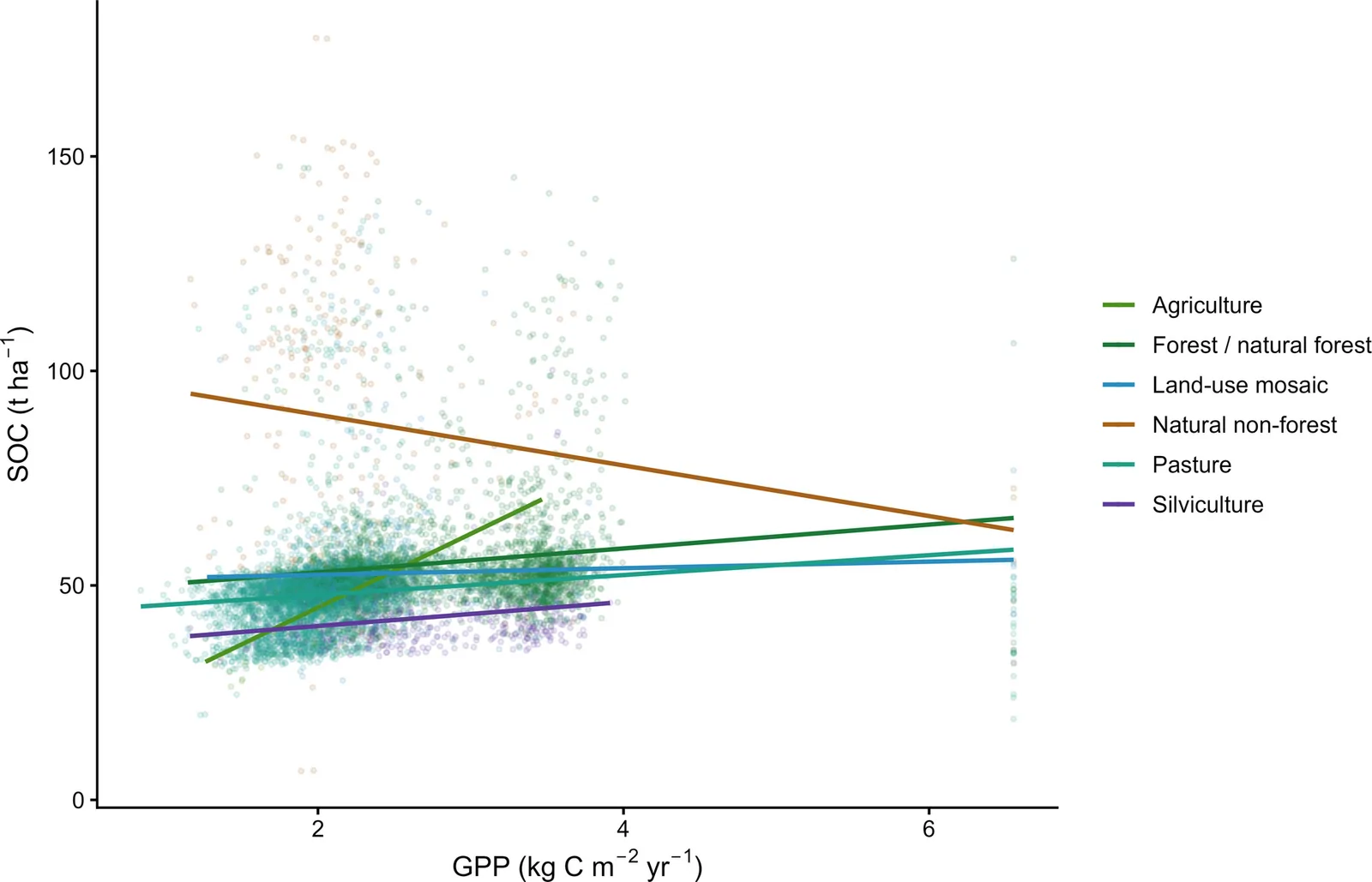
Relationship between gross primary productivity (GPP) and soil organic carbon (SOC) by land use class. Points represent individual observations, and the lines indicate the linear regression models fitted for each land use class

The relationship between GPP and SOC varied considerably among land use classes (Fig. 3, Table 4). Overall, a positive trend was observed, although with limited explanatory power in most land use systems, indicating that current productivity is not the primary determinant of regional-scale soil carbon stocks.

**Table 4:** Linear regression equations describing the relationship between gross primary productivity (GPP) and soil organic carbon (SOC) across land use classes

Land use class	Linear equation	*R*^2^	*p* value
Forest	SOC = 48.320 + 2.652 × GPP	0.014	< 0.001
Natural nonforest formation	SOC = 88.367 − 0.040 × GPP	< 0.001	0.930
Agriculture	SOC = 27.183 + 10.092 × GPP	0.124	< 0.001
Pasture	SOC = 41.940 + 3.050 × GPP	0.014	< 0.001
Land use mosaic	SOC = 47.830 + 1.945 × GPP	0.009	< 0.001
Forestry plantations	SOC = 33.992 + 3.138 × GPP	0.051	< 0.001

The strongest association was observed in agricultural areas (*R*^2^ = 0.124; *p* < 0.001), suggesting a relatively stronger, although still weak, coupling between ecosystem productivity and soil carbon in these systems. In the remaining land use classes, the relationship was considerably weaker (*R*^2^ < 0.06), indicating that variation in current GPP explains only a small proportion of the variation in SOC. In forest ecosystems, for example, the low explanatory power suggests that current productivity alone is insufficient to explain the observed variation in soil carbon stocks, which may also reflect longer-term processes and soil properties.

In natural nonforest formations, the relationship between GPP and SOC was not statistically significant (*p* = 0.93), providing no evidence of a systematic association between the two variables within this land use class. This pattern is further illustrated by the wide data dispersion and contrasting regression slopes shown in Fig. 3, reinforcing that the relationship between productivity and soil carbon varies among land use classes.

The multiple regression models corroborate these patterns. For SOC, both GPP and soil clay content had significant positive effects, highlighting the role of soil texture in stabilizing organic matter. This model explained 38.2% of the observed variability (*R*^2^ = 0.382), indicating satisfactory performance given the complexity of the processes involved. For GPP, land use emerged as the primary explanatory factor, whereas clay content exhibited a weak negative effect. The model explained 37.4% of the observed variability (*R*^2^ = 0.374), reinforcing the importance of vegetation structure and land management in determining ecosystem productivity.

### Temporal dynamics of GPP and SOC

**Fig. 4:**
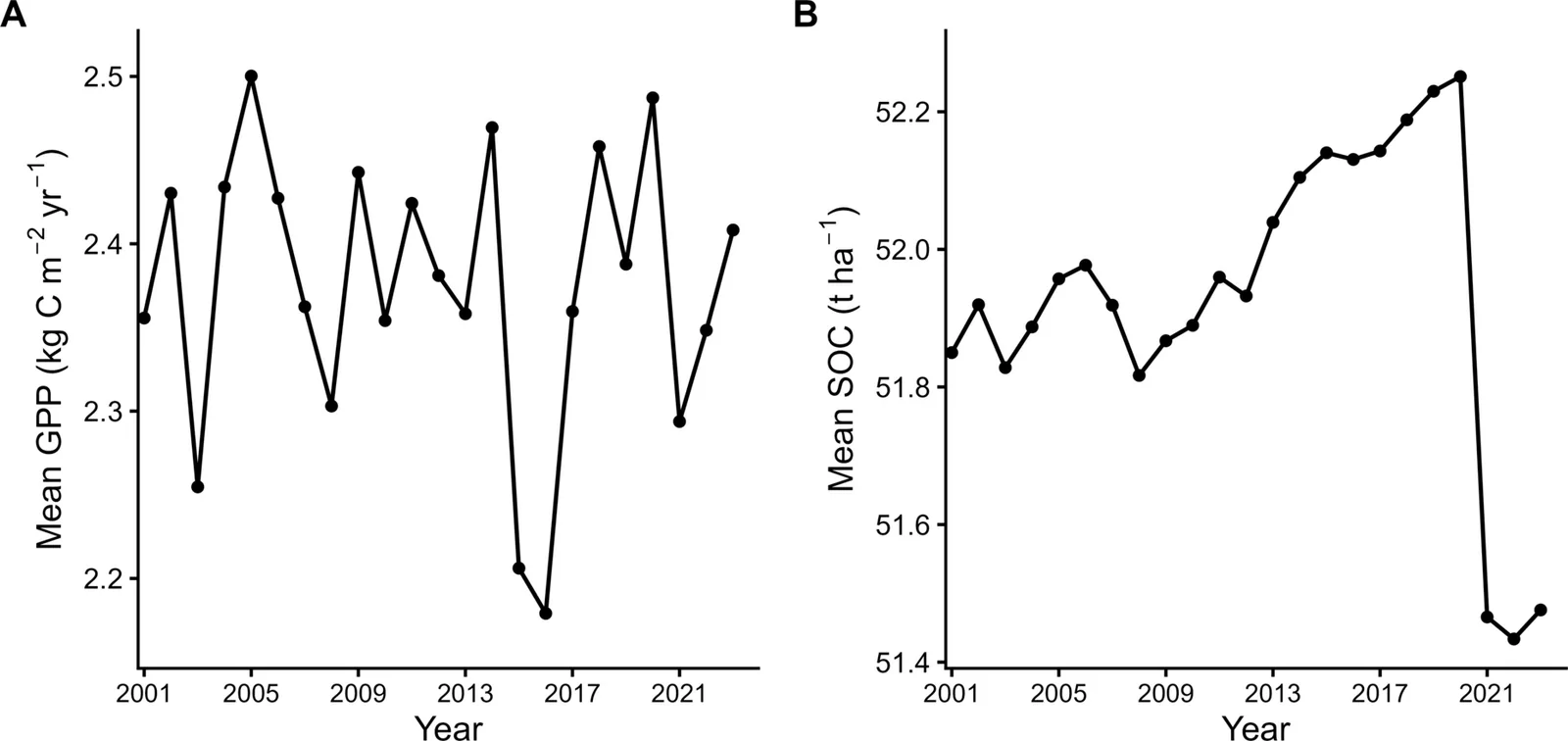
Temporal dynamics of gross primary productivity (GPP) and soil organic carbon (SOC) from 2001 to 2023. **A** Time series of mean GPP by land use class. **B** Time series of mean SOC by land use class

The temporal dynamics of gross primary productivity (GPP) and soil organic carbon (SOC) revealed contrasting patterns between functional stability and gradual changes in soil carbon stocks over the study period (2001–2023) (Fig. 4, Table 5).

**Table 5:** Regional temporal trends in gross primary productivity (GPP) and soil organic carbon (SOC) from 2001 to 2023, showing Kendall’s tau (*τ*) coefficients and statistical significance

Variable	*τ*	*p* value
GPP	−0.059	0.712
SOC	0.304	0.045

At the regional scale, GPP showed no significant temporal trend (*τ* =  − 0.059; *p* = 0.712), being characterized by interannual fluctuations without a consistent directional pattern (Fig. 4A). Occasional declines were observed during specific years, followed by subsequent increases, reflecting interannual variability in ecosystem productivity at the regional scale.

Despite the absence of an overall temporal trend, the analysis by land use class revealed distinct patterns. Natural forests maintained high and relatively stable GPP values throughout the study period, with limited interannual variation in the analyzed data. In contrast, forestry plantations exhibited greater temporal variability, with more pronounced fluctuations and a significant negative trend (*τ* =  − 0.644; *p* < 0.001). Agriculture and pasture displayed lower GPP values, moderate temporal variation, and no significant trends, whereas the land use mosaic showed an intermediate pattern.

SOC exhibited a different temporal behavior (Fig. 4B). At the regional scale, a weak but statistically significant positive trend was detected (*τ* = 0.304; *p* = 0.045), indicating a gradual increase in soil carbon stocks over the study period, particularly until the end of the 2010s. However, this increase was followed by a decline during the final years of the time series, suggesting a possible shift in the system’s trajectory.

The analysis by land use class revealed heterogeneous dynamics that were not captured by the aggregated regional trend. Significant declines in soil carbon stocks were observed in agriculture, forestry plantations, and natural nonforest formations, whereas pasture exhibited a significant positive trend. Natural forests showed a marginal increase, while the land use mosaic remained statistically stable.

Overall, the results indicate a temporal decoupling between ecosystem productivity and soil carbon storage. While GPP showed no significant directional trend throughout the study period, SOC exhibited a slower and more cumulative temporal pattern, consistent with the influence of long-term processes such as organic matter cycling, soil management, and land use change. This pattern demonstrates that changes in soil carbon stocks may occur even in the absence of significant changes in ecosystem productivity, highlighting the importance of integrated temporal analyses for understanding the dynamics of ecosystem services.

### Spatial patterns and functional hotspot regimes of GPP and SOC

**Fig. 5:**
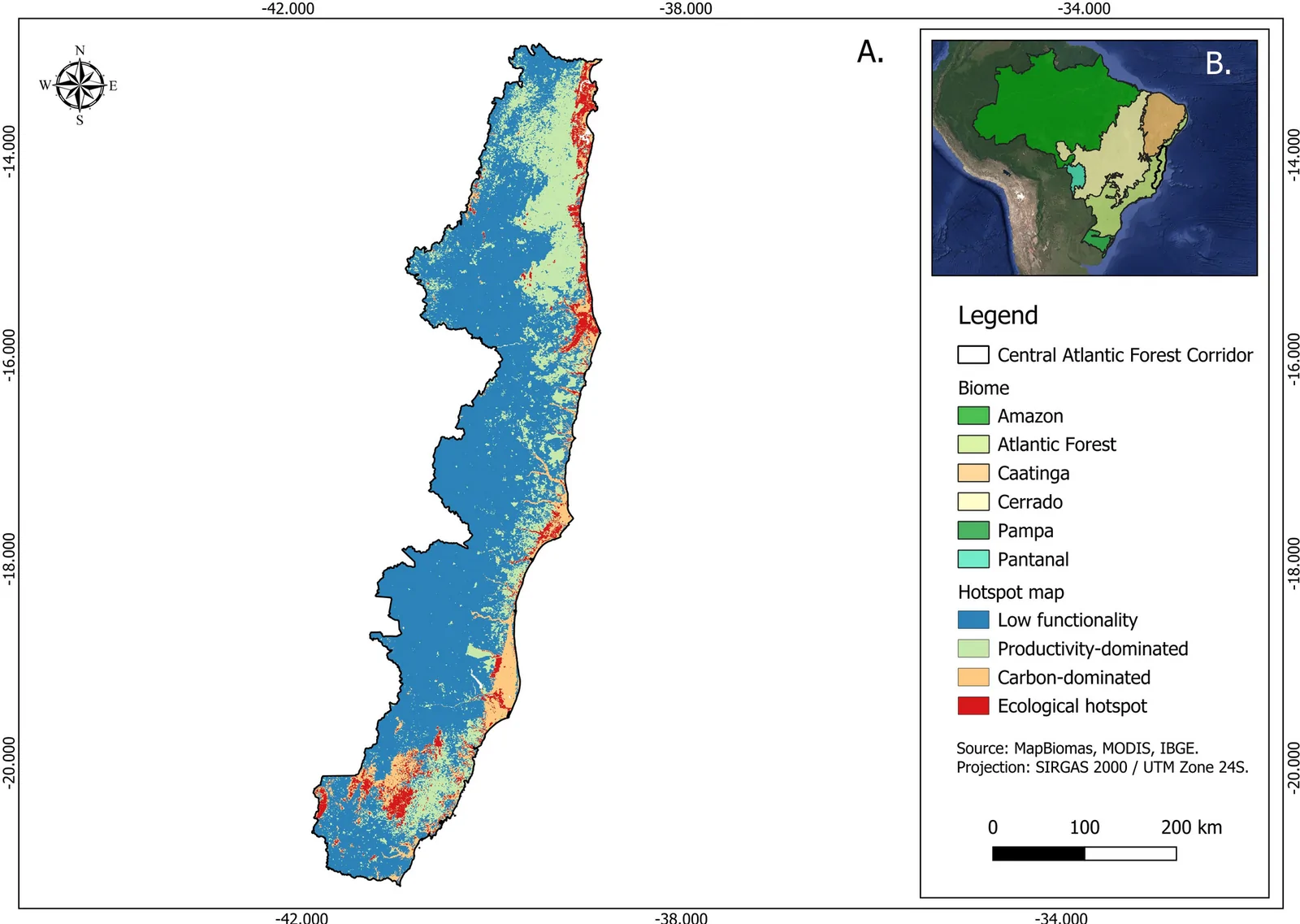
Spatial distribution of GPP and SOC functional hotspots in the Central Atlantic Forest Corridor (CAFC), Brazil. **A** Functional regimes derived from the integration of GPP and SOC. **B** Location of the CAFC within the Brazilian biomes

The integrated spatial analysis of gross primary productivity (GPP) and soil organic carbon (SOC) identified four distinct functional regimes across the landscape, exhibiting a highly heterogeneous spatial distribution (Fig. 5).

The ecological hotspot class (high SOC and high GPP) exhibited a spatially clustered distribution, forming continuous or semicontinuous patches strongly associated with forest areas, which accounted for approximately 66.9% of its occurrence. These patterns identify regions where high biomass production and substantial soil carbon accumulation converge.

In contrast, the low functionality class (low SOC and low GPP) was widely distributed across the study area, predominating in the inland portions of the landscape. This class was strongly dominated by pasture, which accounted for approximately 82.2% of all observations, forming the principal landscape matrix.

The carbon-dominated class (high SOC and low GPP) was also strongly associated with pasture (60.5%), exhibiting a relatively widespread distribution, although less continuous than that observed for the low functionality class. In contrast, the productivity-dominated class (high GPP and low SOC) displayed a more heterogeneous spatial distribution, with a greater contribution from forests (37.4%) and forestry plantations (20.7%), indicating distinct combinations of primary productivity and soil carbon storage.

Overall, the spatial configuration of the functional regimes was characterized by a mosaic pattern, in which highly functional ecological hotspots were embedded within a matrix of lower ecosystem performance, with transitions mediated by the intermediate functional classes. This arrangement highlights the pronounced spatial variability in the relationship between GPP and SOC.

**Fig. 6:**
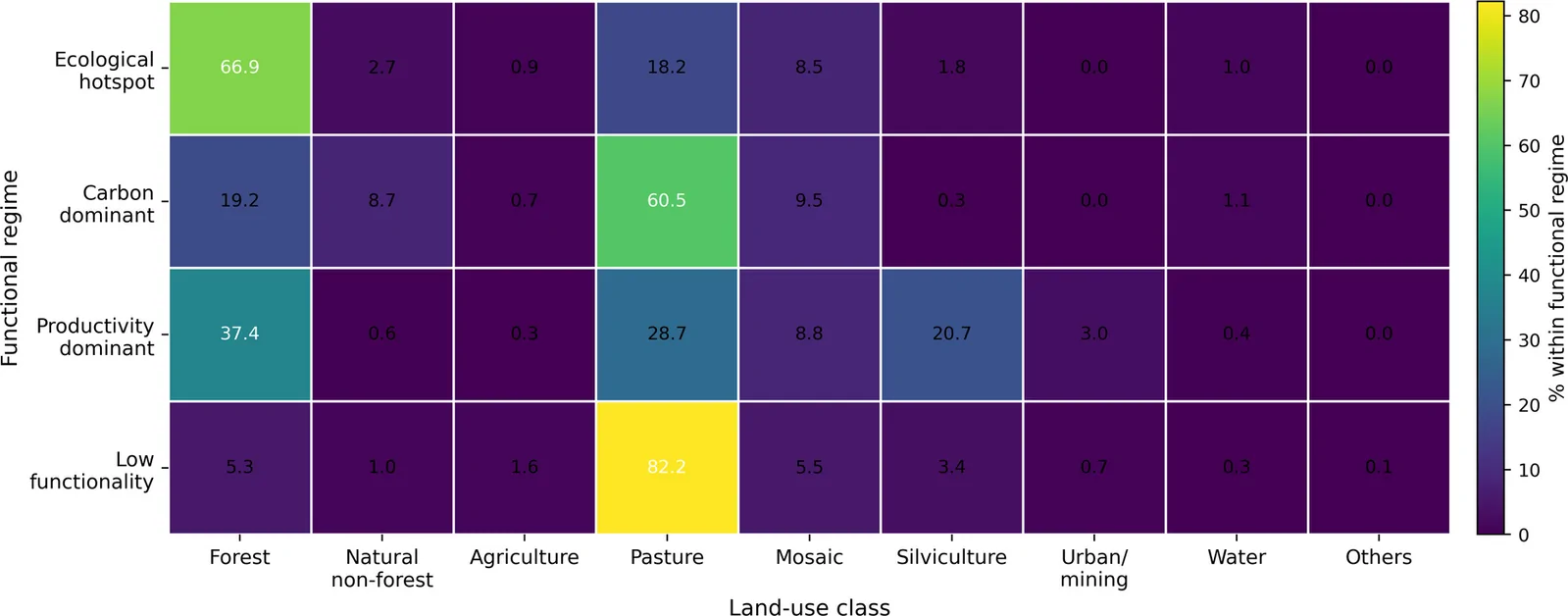
Percentage distribution of the functional regimes resulting from the combination of gross primary productivity (GPP) and soil organic carbon (SOC) across land use and land cover classes. Values represent the proportion (%) of each land use class within each functional regime

The distribution of functional regimes across land use classes, summarized in Fig. 6 (heatmap), revealed contrasting patterns of association between ecosystem productivity and soil carbon storage. This relationship was highly significant (*χ*^2^ = 170,350.91; df = 24; *p* < 0.001), indicating that the occurrence of the different functional regimes was not independent of land use.

### Spatial autocorrelation and spatial regression

Both GPP and SOC exhibited pronounced spatial dependence throughout the 2001–2023 period (Table 6). Global Moran’s *I* for GPP averaged 0.578, with annual values ranging from 0.512 to 0.656, whereas SOC showed stronger and more temporally stable spatial autocorrelation, with a mean Moran’s *I* of 0.704 and annual values ranging from 0.703 to 0.706. These results indicate persistent positive spatial clustering of ecosystem productivity and soil carbon stocks across the Central Atlantic Forest Corridor.

**Table 6:** Spatial autocorrelation and spatial error model (SEM) diagnostics for gross primary productivity (GPP) and soil organic carbon (SOC) across the Central Atlantic Forest Corridor during 2001–2023

Response variable	Raw Moran’s *I*	OLS residual Moran’s *I*	SEM *λ*	SEM residual Moran’s *I*	Mean AIC OLS	Mean AIC SEM
GPP	0.578 (0.512–0.656)	0.302 (0.238–0.358)	0.727 (0.663–0.787)	− 0.034 (− 0.042 to − 0.025)	21,861.2	17,132.5
SOC	0.704 (0.703–0.706)	0.596 (0.576–0.612)	0.884 (0.882–0.886)	− 0.018 (− 0.019 to − 0.016)	113,957.6	99,719.8

Values represent annual means for 2001–2023, with minimum–maximum values in parentheses. Moran’s *I* was calculated using a row-standardized 8-nearest-neighbor spatial weight matrix. *λ* represents the spatial error coefficient estimated by the SEM

Spatial dependence remained evident after accounting for the explanatory variables included in the conventional multiple linear regression models. Residual Moran’s *I* averaged 0.302 for the GPP model (0.238–0.358) and 0.596 for the SOC model (0.576–0.612). The higher residual spatial autocorrelation observed for SOC indicates that GPP, soil clay content, and land use did not fully account for the spatial structure of soil carbon stocks.

The spatial error models (SEMs) substantially reduced the remaining spatial dependence. The spatial error coefficient (*λ*) averaged 0.727 for GPP (0.663–0.787) and 0.884 for SOC (0.882–0.886), indicating a strong spatial component in both models, particularly for SOC. After accounting for spatial dependence, residual Moran’s *I* decreased to − 0.034 for GPP (− 0.042 to − 0.025) and − 0.018 for SOC (− 0.019 to − 0.016), remaining close to zero throughout the analyzed period.

Model comparison also supported the spatial specification. Mean AIC decreased from 21,861.2 in the nonspatial GPP models to 17,132.5 in the corresponding SEM and from 113,957.6 to 99,719.8 for SOC. These reductions, together with the near-zero residual Moran’s *I* values, indicate a substantial improvement in model fit after accounting for spatial dependence.

Importantly, controlling for spatial dependence altered the estimated relationship between GPP and SOC. In the SOC spatial models, the GPP coefficient averaged − 0.030, ranging from − 0.432 to 0.224 across the study period, and was statistically significant in only 3 of the 23 analyzed years. Thus, the relationship between current ecosystem productivity and soil carbon stocks was weak and temporally inconsistent after accounting for spatial structure.

## Discussion

### Land use as the primary driver of GPP–SOC divergence

The results of this study demonstrate that the interaction between gross primary productivity (GPP) and soil organic carbon (SOC) is strongly structured by land use and landscape organization, revealing the existence of distinct functional regimes within the Central Atlantic Forest Corridor. GPP represents the largest carbon flux in terrestrial ecosystems and is a key variable for understanding the regulation of the global carbon cycle, the impacts of climate change, and the mitigation potential of ecosystems (Friedlingstein et al., 2026; Liao et al., 2023; Zhu et al., 2024).

Our findings show that land use exerts significant control over both GPP and SOC, although through different mechanisms. Whereas GPP responds rapidly to vegetation structure, leaf area, absorbed radiation, water availability, and photosynthetic efficiency, SOC reflects long-term cumulative processes governed by soil texture, mineralogy, moisture, aggregation, organo-mineral stabilization, and land use history (Cotrufo et al., 2019; Lavallee et al., 2020; Liao et al., 2023). This distinction helps explain the weak coupling observed between vegetation productivity and soil carbon stocks across the analyzed land use classes. Although some GPP–SOC relationships were statistically significant, their generally low explanatory power indicates that statistical significance should not be interpreted as evidence of a strong ecological dependence between these variables.

The highest GPP values recorded in forest ecosystems are consistent with global studies identifying forests as the most productive land cover type across most environmentally comparable regions. Krause et al. (2022) estimated that forests exhibit higher mean GPP than both pastures and croplands across approximately two-thirds of comparable landscapes, while also showing that historical agricultural expansion has reduced global GPP by approximately 4.4%. These findings reinforce the conclusion that the conversion of forests into anthropogenic land uses generally reduces landscape photosynthetic capacity and annual carbon assimilation (Krause et al., 2022).

Within the Atlantic Forest, the high GPP observed in native forest formations confirms the importance of remaining forest fragments for ecosystem carbon assimilation. GPP dynamics in the Atlantic Forest can exhibit marked seasonal variation; for example, a study conducted in Itatiaia National Park reported higher GPP during the rainy season and lower values during the dry season (Santana et al., 2025). In the present study, however, the consistently high and relatively stable GPP observed in forest formations across the Central Atlantic Forest Corridor indicates the maintenance of comparatively high ecosystem productivity throughout the analyzed period, despite the highly fragmented configuration of the regional landscape.

However, the intermediate SOC values observed in forest ecosystems, despite their high productivity, indicate that soil carbon storage is not determined exclusively by current ecosystem productivity. Although greater GPP can increase the input of litter, fine roots, and root exudates, long-term carbon persistence depends primarily on physicochemical stabilization, aggregate protection, and the association of organic matter with soil minerals (Cotrufo et al., 2019; Lehmann & Kleber, 2015; Schmidt et al., 2011). Consequently, highly productive ecosystems do not necessarily contain the largest SOC stocks, particularly where historical disturbance, erosion, soil limitations, or rapid organic matter decomposition have occurred.

### Subsurface carbon accumulation in open natural ecosystems

The high SOC stocks observed in natural nonforest formations, combined with relatively low GPP and the absence of a statistically significant relationship between these variables, represent one of the most ecologically relevant findings of this study. This pattern indicates that high soil carbon storage can occur independently of contemporary vegetation productivity. Tropical grasslands, savannas, and other open ecosystems can maintain substantial belowground carbon stocks and should not be interpreted as degraded systems solely because of their comparatively low aboveground biomass or tree cover (Veldman et al., 2015). The persistence of SOC, in turn, is influenced by processes such as microbial transformation, aggregate protection, and organo-mineral associations that stabilize organic matter within the soil matrix (Cotrufo et al., 2019; Lavallee et al., 2020). Therefore, the high SOC stocks observed in natural nonforest formations should not be attributed to a single mechanism but rather interpreted as evidence of the importance of belowground carbon storage and soil-mediated carbon persistence in these ecosystems.

These findings have important implications for conservation assessments. Approaches focused primarily on vegetation greenness, productivity, or aboveground biomass may not fully capture the carbon storage importance of natural nonforest ecosystems. Within the Central Atlantic Forest Corridor, the combination of comparatively low GPP and high SOC highlights the importance of considering soil carbon alongside vegetation-based indicators when evaluating ecosystem carbon functions and developing conservation strategies.

### Agricultural systems and the coupling between GPP and SOC

In agricultural systems, the relatively stronger relationship between GPP and SOC observed in the nonspatial analysis may reflect the more direct influence of land management on both variables simultaneously. Practices such as crop rotation, permanent soil cover, no-till farming, and crop residue incorporation can simultaneously influence ecosystem productivity and soil carbon stocks, whereas intensive soil disturbance may accelerate carbon losses through erosion and organic matter mineralization (Minasny et al., 2017; Paustian et al., 2016). The substantial within-class variability observed for agricultural SOC may therefore reflect differences in management intensity, cropping systems, soil properties, and land use history across the regional agricultural landscape.

However, the spatial analysis showed that the apparent coupling between GPP and SOC requires cautious interpretation. Both variables exhibited pronounced positive spatial autocorrelation, with stronger spatial dependence for SOC than for GPP. Substantial spatial dependence also remained in the residuals of the conventional models, particularly for SOC, indicating that the explanatory variables did not fully capture the spatial organization of soil carbon stocks. The spatial error models substantially reduced this residual dependence, with Moran’s *I* approaching zero after spatial correction. More importantly, after accounting for spatial structure, the effect of GPP on SOC was weak and temporally inconsistent, being statistically significant in only 3 of the 23 analyzed years. This indicates that part of the association detected by conventional regressions reflected shared spatial structure rather than a consistently strong ecological relationship, reinforcing the broader decoupling between contemporary ecosystem productivity and soil carbon storage (Bivand & Wong, 2018; Dormann et al., 2007).

### Temporal decoupling and the trajectory of soil carbon

The temporal stability of regional GPP should be interpreted with caution. Remote sensing products based on light-use efficiency models, such as MOD17, are widely used but are subject to uncertainties associated with biome-specific parameterization, vegetation heterogeneity, and the representation of temporally varying physiological and environmental processes (Liao et al., 2023; Zhang et al., 2023; Zhu et al., 2024). Zhang et al. (2023) demonstrated that temporal scaling of MODIS fAPAR can improve forest GPP estimates and reduce uncertainties, particularly when combined with local optimization of MOD17 model parameters.

This limitation is particularly relevant for the Central Atlantic Forest Corridor, where pronounced landscape heterogeneity includes native forests, cabruca agroforestry systems, pastures, agricultural areas, and forestry plantations. Such heterogeneity may not be fully represented by light-use efficiency models relying on generalized parameterizations. Likewise, the SOC estimates derived from MapBiomas Soil are model-based predictions and therefore incorporate uncertainties associated with field data availability, environmental covariates, and the predictive modeling procedure. In addition, harmonizing datasets with different native spatial resolutions to the 500-m MODIS grid may smooth fine-scale spatial variability and generate mixed pixels, particularly in highly fragmented landscapes. Consequently, the results should be interpreted primarily as regional-scale patterns rather than as estimates of local conditions.

Because the annual SOC estimates are derived from model-based predictions rather than repeated field measurements, the temporal trends identified here should be interpreted as trends in estimated SOC rather than direct evidence of measured changes in soil carbon stocks. The weak but significant positive regional trend in SOC indicates a gradual increase in estimated soil carbon stocks during the study period, although this pattern was not homogeneous across land use classes. Declining trends were observed in agriculture, forestry plantations, and natural nonforest formations, whereas pasture showed a positive trend. Because the present analysis did not explicitly incorporate management history, land use transitions, soil disturbance, or climatic and hydrological drivers, the mechanisms underlying these contrasting trajectories cannot be determined from the available data. Previous studies nevertheless demonstrate that SOC responses to land use change are strongly influenced by land use transitions, time since conversion, and subsequent management practices (Bossio et al., 2020; Don et al., 2011; Poeplau et al., 2011). The contrast between the temporal stability of GPP and the heterogeneous trajectories of SOC therefore reinforces the temporal decoupling between contemporary vegetation productivity and soil carbon stocks and highlights the importance of long-term integrated monitoring of both variables.

### Functional regimes and landscape management

The spatial integration of GPP and SOC revealed distinct functional regimes across the Central Atlantic Forest Corridor. Ecological hotspots, characterized by the coexistence of high GPP and high SOC, were predominantly associated with forest ecosystems, highlighting the importance of native forest remnants for simultaneously maintaining ecosystem productivity and soil carbon stocks. This pattern is consistent with the broader role of Atlantic Forest conservation and restoration in maintaining biodiversity, carbon storage, and ecosystem services (Brancalion et al., 2019; Krause et al., 2022; Strassburg et al., 2020).

In contrast, low-functionality areas, characterized by low GPP and low SOC, were predominantly associated with pasturelands. The high representation of pastures within this regime indicates that these areas combine comparatively low vegetation productivity with low soil carbon stocks at the regional scale. Pasture degradation and management practices can substantially influence SOC stocks, with degraded pastures generally showing lower soil carbon stocks and recovered or improved management systems presenting potential for SOC accumulation (Oliveira et al., 2022). These results therefore identify pasture-dominated areas as relevant candidates for restoration and improved land management within the Central Atlantic Forest Corridor, although site-specific assessments are required to establish restoration priorities.

The intermediate regimes further demonstrate that the relationship between ecosystem productivity and soil carbon storage is neither linear nor spatially uniform. Productivity-dominated areas combine high GPP with comparatively low SOC, whereas carbon-dominated areas exhibit the opposite pattern. Rather than attributing these combinations to specific mechanisms not directly evaluated in this study, these regimes should be interpreted as evidence that vegetation productivity and soil carbon storage respond to partially independent environmental and land use controls. This interpretation is also consistent with the weak and temporally inconsistent GPP–SOC relationship observed after accounting for spatial dependence.

The strong statistical association between functional regimes and land use further demonstrates that their distribution is closely related to landscape composition. From an applied perspective, the combined GPP–SOC classification provides a spatial framework for distinguishing areas where high ecosystem productivity and soil carbon stocks coexist from areas where one or both functions are comparatively reduced. Such information can complement existing criteria used in conservation, ecological restoration, and sustainable land management (Brancalion et al., 2019; Strassburg et al., 2020).

Within the Central Atlantic Forest Corridor, the results support two complementary management directions: maintaining forest-dominated areas where high GPP and high SOC coincide and improving management or restoration in areas characterized by simultaneously low values of both variables, particularly pasture-dominated landscapes. However, the functional regimes identified here should be interpreted as indicators of relative ecological conditions rather than direct measures of ecosystem integrity or restoration priority. Integrating these spatial patterns with biodiversity, climate, hydrological, socioeconomic, and restoration-feasibility information could provide a more comprehensive basis for future territorial planning.

## Conclusions

The results demonstrate that the interaction between gross primary productivity (GPP) and soil organic carbon (SOC) is strongly associated with land use and land cover, resulting in distinct functional regimes across the Central Atlantic Forest Corridor. The integrated analysis of these variables revealed spatial and ecological patterns that would not have been identified through separate assessments.

GPP exhibited the highest values in forest ecosystems, whereas SOC showed distinct patterns among land use classes and was also associated with soil properties. The generally weak relationship between GPP and SOC indicates a functional decoupling between contemporary ecosystem productivity and soil carbon storage. This interpretation was strengthened after accounting for spatial dependence, as the effect of GPP on SOC became weak and temporally inconsistent, while the spatial error models substantially reduced the residual spatial autocorrelation. These findings demonstrate that high vegetation productivity does not necessarily correspond to high soil carbon stocks at the landscape scale.

The bivariate classification distinguished four functional regimes: low functionality, productivity-dominated, carbon-dominated, and ecological hotspots. Ecological hotspots occurred predominantly in forest ecosystems, whereas low-functionality regimes were strongly associated with pasturelands. These spatial patterns highlight the importance of maintaining forest ecosystems while also identifying pasture-dominated areas where improved management and ecological restoration may contribute to enhancing ecosystem functions.

Overall, the integration of GPP and SOC provides a spatially explicit framework for evaluating complementary dimensions of ecosystem carbon functioning in heterogeneous tropical landscapes. Rather than defining conservation or restoration priorities in isolation, this approach can complement biodiversity, climatic, hydrological, and socioeconomic information to support territorial planning, ecological restoration, and carbon-oriented environmental policies.

## Data Availability

No datasets were generated or analysed during the current study.

## Code availability

The scripts and codes used for spatial processing and statistical analyses are available from the corresponding author upon reasonable request.
